# Timing of Antimicrobial Lock Replacement for Gram-Positive Port Infections: Results of a Randomized Trial

**DOI:** 10.3390/antibiotics15020157

**Published:** 2026-02-02

**Authors:** César Bustos, José R. Yuste, Aitziber Aguinaga, Asunción Parra, Francisco Carmona-Torre, José R. Azanza, Carlos Lacasa, José L. Del Pozo

**Affiliations:** 1 Division of Infectious Diseases, Clínica Universidad de Navarra, 31008 Pamplona, Spain; cbustosg@uandes.cl (C.B.);; 2 Department of Clinical Microbiology, Clínica Universidad de Navarra, 31008 Pamplona, Spain; 3 Department of Internal Medicine, Clínica Universidad de Navarra, 31008 Pamplona, Spain; 4 Navarra Institute for Health Research (IdiSNA), 31008 Pamplona, Spain; 5 Department of Clinical Pharmacology, Clínica Universidad de Navarra, 31008 Pamplona, Spain; 6 Biogipuzkoa HRI, Infectious Diseases Area, Infectious Diseases and Global Health Research Group, 20014 San Sebastian, Spain; 7 Department of Pharmacy, Clínica Universidad de Navarra, 31008 Pamplona, Spain

**Keywords:** catheters, ports, implantable, bacteremia, indwelling, anti-infective agents, vancomycin, tigecycline, linezolid, daptomycin, teicoplanin

## Abstract

**Background**: Conservative management of port-related bacteremia often includes locally administered antimicrobials, known as antimicrobial lock therapy (ALT). Current guidelines recommend daily replacement of antimicrobial lock solutions (ALSs). We aimed to evaluate whether ALSs could remain effective with extended dwell times of up to 10 days. **Methods**: In this randomized clinical trial, patients with noninfected, recently implanted ports were assigned to one of five ALS dwell-time groups, ranging from 1 to 10 days. Each ALS contained heparin plus an antimicrobial at standard intraluminal concentrations: vancomycin 2 mg/mL, teicoplanin 10 mg/mL, linezolid 1.8 mg/mL, daptomycin 5 mg/mL, or tigecycline 4.5 mg/mL. The primary endpoint was the time at which intraluminal drug concentrations decreased below 1 mg/mL (ClinicalTrials.gov NCT01592032). **Results**: Vancomycin and linezolid concentrations fell significantly below 1 mg/mL after 3 days of dwell time. Daptomycin and tigecycline concentrations decreased significantly after 7 days but remained above 1 mg/mL. Teicoplanin concentrations did not decline significantly after 7 days. **Conclusions**: Optimal ALS dwell time depends on the antimicrobial agent. Vancomycin and linezolid locks require daily replacement, whereas daptomycin, tigecycline, and teicoplanin locks maintain therapeutic concentrations for up to 7 days. These findings support individualized ALS replacement strategies, potentially reducing the need for daily interventions.

## 1. Background

Totally implantable venous access ports are widely used in cancer patients, but their use is frequently complicated by thrombosis and infection [1]. The risk of catheter-related bloodstream infection (BSI) is estimated at 0.1 per 1000 port-days [2], and repeated device manipulation further increases this risk [3]. Overall, up to 10% of port carriers will experience device-related infectious complications [4], and more than half of these cases ultimately require port removal [5]. Staphylococci account for nearly 70% of all port-related BSIs [6]. Despite their relatively low incidence, port-related infections are associated with significant clinical consequences, including treatment delays, increased healthcare utilization, and excess morbidity, particularly in patients receiving long-term chemotherapy or parenteral therapies. As a result, strategies aimed at preventing device removal while ensuring infection control have become a priority in modern oncological care. Management of uncomplicated catheter-related bacteremia (CRB) typically combines systemic therapy with local antimicrobial administration through antimicrobial lock therapy (ALT). Current recommendations suggest 10–14 days of treatment [7]. ALT involves instilling a highly concentrated antimicrobial solution into the catheter lumen and maintaining it for a defined dwell time to eradicate intraluminal microorganisms [8]. According to the Infectious Diseases Society of America (IDSA), antimicrobial lock solutions (ALSs) should generally be replaced every 24 h, except in the case of hemodialysis catheters [7]. However, evidence is lacking to determine the optimal replacement interval for ALSs. Over the last decade, antimicrobial lock therapy has been increasingly adopted as an adjunctive strategy for catheter salvage, supported by growing clinical experience and observational evidence. Recent reviews highlight substantial heterogeneity in antimicrobial agents, concentrations, dwell times, and replacement intervals used across institutions, underscoring the absence of robust pharmacokinetic or outcome-driven data to guide standardized practice. In particular, current recommendations regarding daily lock replacement are largely extrapolated from expert opinion or hemodialysis catheter models, rather than from direct pharmacokinetic evaluation in totally implantable ports.

Extending ALS dwell time could reduce port manipulation to only once or twice during the course of therapy, thereby lowering costs and minimizing procedure-related morbidity without compromising efficacy. Encouraged by previous reports and by our own clinical experience [9,10,11], we designed a randomized clinical trial to evaluate the optimal replacement intervals for five commonly used ALSs—vancomycin, teicoplanin, linezolid, daptomycin, and tigecycline—instilled into noninfected ports and maintained in situ for variable dwell times. These agents were selected because they represent the antimicrobials most frequently used in clinical practice for the management of suspected or confirmed Gram-positive port-related bloodstream infections, particularly in oncology and long-term catheter settings. In addition, they encompass a broad range of physicochemical and pharmacokinetic properties—including molecular weight, protein binding, lipophilicity, and chemical stability—which may differentially influence intraluminal concentration decay and lock performance. Previous clinical and experimental studies have reported heterogeneous results with these agents in antimicrobial lock therapy, highlighting the need for comparative pharmacokinetic characterization. Beyond clinical heterogeneity, antimicrobial lock therapy raises important pharmacokinetic and antimicrobial stewardship considerations. Prolonged exposure to high intraluminal concentrations may enhance efficacy against intraluminal microorganisms, but insufficiently characterized dwell times could also result in prolonged exposure to sub-inhibitory concentrations, potentially favoring resistance selection. Recent clinical and translational studies emphasize the need to balance pharmacokinetic adequacy with ecological impact when designing lock strategies. In this context, pharmacokinetic-driven studies that directly measure intraluminal antimicrobial concentrations over time represent a critical step toward rational optimization of antimicrobial lock therapy. By characterizing concentration decay patterns under controlled conditions, such studies may inform the design of future outcome-driven trials and support individualized lock replacement strategies tailored to specific antimicrobial agents.

## 2. Results

**Figure 1:**
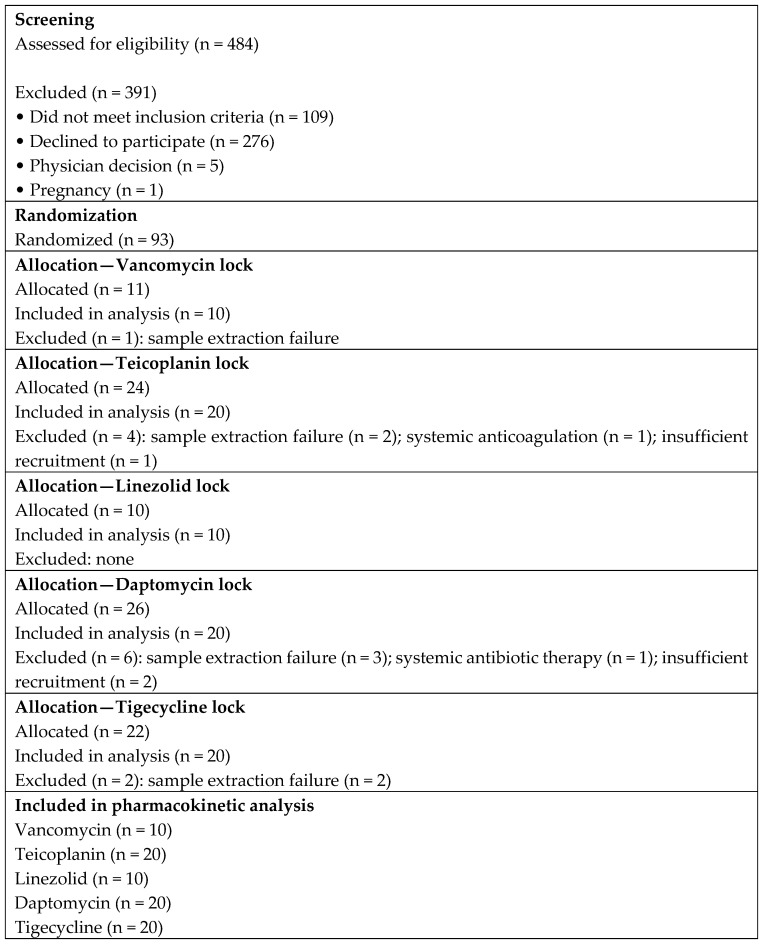
Flow diagram of patient screening, randomization, allocation, and inclusion in the pharmacokinetic analysis. A total of 484 patients with newly implanted venous access ports were assessed for eligibility, of whom 93 were randomized to receive one of five antimicrobial lock solutions. Exclusions after randomization were mainly due to sample extraction failure, protocol deviations, or insufficient recruitment for specific dwell-time intervals.

Over the 32-month study period, 484 consecutive patients were screened for eligibility (Figure 1). Of the 484 patients assessed for eligibility, 391 were excluded primarily due to failure to meet inclusion criteria or refusal to participate. Additional exclusions after randomization were mainly related to sample extraction failure, insufficient recruitment for specific dwell-time intervals, or protocol deviations. A total of 93 patients were randomized into the five ALS groups: 11 to vancomycin, 24 to teicoplanin, 10 to linezolid, 26 to daptomycin, and 22 to tigecycline. Baseline characteristics, including sex, age, underlying malignancy, and mean time from catheter placement to study entry, were comparable across groups (Table 1). No port-related infections occurred in any of the patients during the study, and no serious drug-related adverse events were observed.

After 1 day of dwell time, all five ALSs maintained antimicrobial concentrations above 1 mg/mL. At 3 days, the median concentrations of vancomycin and linezolid had fallen below 1 mg/mL, leading to termination of further randomization in these groups. The decrease in concentrations of both drugs compared with baseline was statistically significant (*p* = 0.043).

Patients continued to be enrolled in the teicoplanin, daptomycin, and tigecycline groups for dwell times of 5 and 7 days. Teicoplanin median concentrations remained above 1 mg/mL at both time points, with no significant difference compared with the instilled concentration (*p* = 0.893). In contrast, daptomycin and tigecycline concentrations decreased significantly after 7 days (*p* = 0.043) but still remained above 1 mg/mL (Table 2).

Due to limited enrollment, the 10-day dwell time group could not be completed, and the study concluded without data for this interval.

## 3. Discussion

Antimicrobial lock replacement should be tailored to the agent: vancomycin and linezolid require daily changes, while teicoplanin, daptomycin, and tigecycline remain effective for up to 7 days, reducing interventions without compromising efficacy. No conclusions regarding comparative clinical efficacy or therapeutic equivalence between agents can be drawn from the present study. This agent-specific behavior underscores the limitations of uniform replacement strategies and highlights the importance of integrating pharmacokinetic principles into antimicrobial lock management rather than relying on fixed, one-size-fits-all recommendations.

The Infectious Diseases Society of America (IDSA) guidelines recommend that the dwell time of an antimicrobial lock solution (ALS) should not exceed 24 h, except for hemodialysis catheters [7]. Nevertheless, the optimal replacement interval remains undefined, with reported recommendations ranging between 8 and 24 h per day [12]. Our trial demonstrates that the decline in antimicrobial concentration within ALS instilled into ports is critical for determining replacement time. Consequently, the general recommendation to replace ALS every 24 h should be tailored according to the antimicrobial agent employed. Specifically, ALSs containing teicoplanin (10 mg/mL), daptomycin (5 mg/mL), or tigecycline (4.5 mg/mL) could be safely replaced every 7 days, whereas ALSs with vancomycin or linezolid should be replaced daily. Although initial antimicrobial concentration is an important determinant of exposure, the present study was designed to evaluate concentration preservation kinetics over time rather than to compare absolute drug exposure across agents. Differences in optimal dwell time should therefore be interpreted as reflecting agent-specific pharmacokinetic stability profiles within the lock environment, rather than baseline concentration alone. Accordingly, these findings should be interpreted within a pharmacokinetic framework, emphasizing intraluminal concentration stability rather than assumptions of sustained antimicrobial activity or clinical equivalence

The duration of ALS exposure to the catheter lumen is pivotal for biofilm eradication. Our study was designed to establish the longest effective dwell time without compromising intraluminal concentrations. Extrapolating findings from hemodialysis catheter–related bacteremia (CRB) to port-related infections is problematic, as hemodialysis catheters are accessed every 48–72 h, whereas ports are generally used daily or intermittently for chemotherapy administration. It is important to note that the concept of half-life in antimicrobial lock solutions differs fundamentally from systemic pharmacokinetic half-life. In the lock setting, effective half-life is primarily determined by chemical stability, adsorption to catheter materials, dilution effects, and diffusion dynamics rather than by metabolic clearance. These factors jointly define concentration decay over time and ultimately inform optimal dwell duration. This distinction is particularly relevant in totally implantable ports, where prolonged intraluminal stasis and limited fluid exchange may amplify the impact of physicochemical stability over classical systemic pharmacokinetic parameters

Several factors influence antimicrobial concentrations and activity [13], including catheter design [14], intrinsic drug properties [15], protein-binding capacity [16], free drug fraction [17], and degradation rate [18]. Fluid dynamics also contribute: the Hagen–Poiseuille velocity distribution for flow [19], passive diffusion into the bloodstream [20], density differences between the lock solution and blood [21], and exchange driven by postural changes [22]. While these mechanisms are well described individually, their combined in vivo impact on intraluminal antimicrobial decay remains insufficiently characterized, reinforcing the need for direct pharmacokinetic measurements under clinical conditions.

Soriano et al. described a gradient of vancomycin concentrations along hemodialysis catheters [23], inferred from sequential aspiration of three blood samples. However, this indirect approach is limited by assumptions regarding catheter segment representation. By contrast, our study analyzed a single 2 mL aspirate from five independent patients per ALS and dwell time, thereby reducing reliance on sequential sampling. Still, we recognize that recommendations based on the median of only five samples per group are limited. As such, the present data should be viewed as providing pharmacokinetic signals rather than definitive concentration–time profiles.

Deposits accumulating in the port reservoir may contribute to the gradual decline of intraluminal concentrations. To date, no in vivo studies have characterized antimicrobial loss in port-locked solutions. This knowledge gap has reinforced the practice of daily ALS replacement, rather than exploring the benefits of instilling maximal concentrations and allowing prolonged dwell times, provided safety and efficacy are maintained. Key unanswered questions remain: would increasing antimicrobial concentrations be safer than extending dwell time, or could higher concentrations enable longer dwell intervals? Addressing these questions will require carefully designed studies that integrate pharmacokinetic monitoring with clinical and microbiological endpoints.

Previous studies have reported success with dwell times beyond 24 h. Sánchez-Muñoz et al. described an 85.7% clinical success rate in 14 patients treated with a 3-day lock combining heparin and vancomycin or amikacin [24], though without measuring residual concentrations. Conversely, Haimi-Cohen et al. found a residual vancomycin concentration of 0.13 mg/mL even 28 days after instillation [25]. While maintaining residual drug levels is relevant, avoiding prolonged exposure to subtherapeutic concentrations is paramount, given the risk of resistance development [26]. Indeed, low vancomycin concentrations can enhance staphylococcal biofilm density [27]. In our trial, we established a conservative threshold of 1 mg/mL, representing at least 1000-fold the MIC for planktonic staphylococci [28] and safely above the mutant prevention concentration [15]. The threshold of 1 mg/mL was selected as a pragmatic pharmacokinetic benchmark based on prior antimicrobial lock literature and pharmacokinetic/pharmacodynamic principles, rather than as a validated in vivo efficacy cutoff. This concentration should therefore not be interpreted as a direct surrogate for clinical effectiveness. Prolonged dwell times may theoretically increase the risk of antimicrobial resistance, particularly if drug concentrations decline into sub-inhibitory ranges. This underscores the importance of defining dwell durations that preserve adequate antimicrobial exposure while avoiding unnecessary prolongation. From an antimicrobial stewardship perspective, pharmacokinetic-informed lock replacement strategies may contribute to minimizing selective pressure while maintaining efficacy. Thus, optimization of dwell time should be viewed not only as a matter of convenience but also as a strategy to mitigate unintended ecological consequences of antimicrobial exposure.

Our findings confirmed a progressive decline in ALS concentrations over time. After 3 days, vancomycin concentrations decreased by 77%, falling below bactericidal thresholds [28]. Adequate antimicrobial levels are essential for targeting persister cells, which tolerate high concentrations despite intrinsic susceptibility, thereby contributing to therapeutic failure and biofilm persistence [29,30,31,32]. Similarly, linezolid concentrations decreased by 60% after 3 days. Clinical data on linezolid as lock therapy remain scarce [10,23], though Sofroniadou et al. reported that linezolid locks prevented CRB in hemodialysis patients [32]. While linezolid may be valuable in specific scenarios—such as prosthetic infections [33] or endocarditis [31,34]—evidence for its use in lock solutions remains limited. These observations further support the need for agent-specific replacement strategies rather than extrapolation across antimicrobial classes.

Teicoplanin emerged as the most stable ALS in our study. Median concentrations remained unchanged after 7 days (*p* = 0.89), possibly due to its affinity for catheter materials [35]. Clinical success rates with daily teicoplanin locks for CRB range from 88% to 100% [11,34]. Our data suggest that this efficacy may derive from the remarkable stability of the 10 mg/mL solution over extended dwell times. This stability profile positions teicoplanin as a particularly suitable candidate for extended dwell strategies in port-related infections.

There is increasing evidence for daptomycin in CRB treatment, both in vivo [25,27,36] and in vitro [28,29]. Experimental catheter infection models with daptomycin 2–5 mg/mL have shown significant eradication of staphylococci [30,31,37,38,39,40,41]. Raad et al. and Meije et al. demonstrated superior outcomes with daptomycin locks compared to glycopeptides [42]. In our trial, daptomycin maintained concentrations > 2000 times the staphylococcal MIC for up to 7 days, supporting extension of replacement intervals to once or twice during the 10–14 days recommended by IDSA [7]. These findings reinforce the pharmacokinetic robustness of daptomycin in the lock setting.

Tigecycline has been rarely used in ALT. Aslam et al. reported an 83% success rate with a lock containing N-acetylcysteine, heparin, and tigecycline 1 mg/mL in hemodialysis CRB [11]. However, tigecycline monotherapy for BSI remains controversial [43]. Minocycline–EDTA locks have demonstrated efficacy in hemodialysis [44] and pediatric oncology patients [45], with dwell times up to 7 days. Our findings suggest that tigecycline may be considered in select scenarios when alternatives are lacking. Nonetheless, further pharmacokinetic and clinical data are needed before routine adoption of tigecycline-based lock strategies.

Overall, we observed that teicoplanin (10 mg/mL), tigecycline (4.5 mg/mL), and daptomycin (5 mg/mL) maintained intraluminal concentrations above 1 mg/mL for 7 days, whereas vancomycin and linezolid did not. We acknowledge the limitations of our study, including small sample sizes and heterogeneity in antimicrobial concentrations. Furthermore, maintaining high intraluminal concentrations for 7 days does not guarantee therapeutic success, given the variability in antimicrobial biofilm penetration [46]. These limitations underscore the exploratory nature of the present findings.

In conclusion, our findings suggest that replacement intervals for ALS in ports could be extended from daily to once every 7 days when using teicoplanin, daptomycin, or tigecycline against coagulase-negative staphylococci (CoNS). By contrast, vancomycin and linezolid locks should continue to be replaced every 24 h. From a translational perspective, the present findings should be viewed as hypothesis-generating and primarily informative for the design of future clinical trials. The pharmacokinetic data provided here may serve to select candidate agents, define lock replacement intervals, and optimize exposure targets in outcome-driven studies specifically powered to evaluate clinical efficacy, microbiological eradication, and catheter salvage rates. By combining a randomized design with detailed pharmacokinetic characterization, this study provides a methodological framework for optimizing antimicrobial lock strategies, representing a necessary step toward subsequent outcome-driven clinical trials. Future studies integrating pharmacokinetic monitoring with clinical outcomes will be essential to confirm the clinical impact of extended dwell strategies suggested by the present work.

## 4. Patients and Methods

### 4.1. Setting and Study Population

This clinical trial was conducted at the University Hospital, Clínica Universidad de Navarra (Pamplona, Spain) between May 2012 and January 2015. All patients with recently implanted venous access ports were eligible. Exclusion criteria were: clinical or microbiological evidence of infection (with or without systemic antimicrobial therapy); known allergy to heparin or any of the study antimicrobials; concomitant systemic anticoagulation; pregnancy; and inability to provide written informed consent. Patients with clinical or microbiological evidence of infection were excluded in order to avoid confounding factors such as established intraluminal biofilm burden, inflammatory exudate, or prior antimicrobial exposure, which could unpredictably affect pharmacokinetic concentration decay and obscure interpretation of intraluminal stability. This selection strategy was intentionally designed to isolate pharmacokinetic behavior within the catheter lumen under controlled, noninfected conditions, allowing assessment of antimicrobial concentration stability independent of host inflammatory responses or ongoing microbial metabolism. The study was approved by the local Institutional Review Board (79/2010) and the Spanish Agency of Medicines and Medical Devices (EudraCT 2010-023814-29). All participants gave written informed consent. The trial was registered at ClinicalTrials.gov (NCT01592032).

### 4.2. Trial Design

Patients were randomly assigned, in a single-blind design, to receive one of five antimicrobial lock solutions (ALSs). Each ALS contained 100 IU/mL of 1% sodium heparin plus one antimicrobial: vancomycin 2 mg/mL, teicoplanin 10 mg/mL, linezolid 1.8 mg/mL, daptomycin 5 mg/mL, or tigecycline 4.5 mg/mL, for a final volume of 10 mL. Antimicrobial concentrations were chosen based on the highest levels reported in the literature [13,25,47] and our clinical experience [9,10,48]. The use of high intraluminal antimicrobial concentrations reflects the established principles of antimicrobial lock therapy, aiming to achieve levels substantially exceeding planktonic minimum inhibitory concentrations and to maximize activity against intraluminal microorganisms. All antimicrobials were reconstituted in normal saline, except linezolid (ready-to-use) and daptomycin (lactated Ringer’s solution). Reconstitution solutions were selected according to manufacturer recommendations and previously published stability data for each antimicrobial. Differences in reconstitution media were therefore dictated by pharmaceutical formulation requirements rather than experimental preference. The heparin concentration of 100 IU/mL was chosen because it reflects standard clinical practice for port locking in many oncology units and is widely used in antimicrobial lock protocols to maintain catheter patency while minimizing bleeding risk. Randomization was computer-generated, and the research team was blinded to antimicrobial assignment. Each patient could participate only once. Laboratory personnel responsible for antimicrobial concentration measurements were blinded to dwell-time allocation. Blinding to the antimicrobial agent was not feasible due to the use of different analytical methods and calibration procedures required for each compound. This partial blinding approach was considered appropriate to preserve analytical accuracy while minimizing potential allocation bias.

ALS dwell times were escalated sequentially (1, 3, 5, 7, and 10 days). Each dwell-time period required inclusion of five patients per antimicrobial group. No formal sample size or power calculation was performed, as the study was conceived as a pharmacokinetic, proof-of-concept trial aimed at characterizing intraluminal concentration decay patterns rather than testing between-agent clinical superiority or non-inferiority. The sample size was determined pragmatically based on feasibility and prior pharmacokinetic lock studies. This stepwise escalation design allowed early termination of specific antimicrobial arms once predefined pharmacokinetic thresholds were crossed, thereby limiting unnecessary patient exposure. Prior to ALS instillation, a 10 mL blood sample was drawn to confirm port sterility. After flushing with 10 mL of 0.9% sodium chloride, 5 mL of ALS was instilled into each port, which then remained untouched until the end of the dwell time. Ports were not accessed during the dwell period in order to avoid dilutional effects and mechanical disturbances that could alter intraluminal drug kinetics. After completion, the first 2 mL withdrawn from the port was analyzed for antimicrobial concentration using high-performance liquid chromatography (HPLC; Agilent Technologies, Santa Clara, CA, USA). Urea concentrations were determined (Synchron^®^ Clinical Systems, Beckman Coulter, Brea, CA, USA), and the systemic-to-sample urea ratio was used to correct antimicrobial concentrations for individual blood dilution [49]. This approach was chosen to standardize sampling across patients and minimize procedural manipulation. Although this strategy may not capture potential concentration gradients along the entire catheter length, it ensured reproducible sampling conditions across all study participants

If the median antimicrobial concentration remained above 1 mg/mL for a given ALS group, a new set of five patients was randomized to the next dwell-time interval. A median concentration < 1 mg/mL terminated randomization for that ALS group. The primary endpoint was the time until intraluminal antimicrobial concentration fell below 1 mg/mL. This predefined pharmacokinetic threshold was selected to provide a conservative benchmark for adequate intraluminal exposure, rather than as a surrogate for clinical efficacy. This study was designed as a proof-of-concept, pharmacokinetic-driven trial and was not powered to detect differences in clinical outcomes or establish superiority between antimicrobial agents.

### 4.3. Statistical Analysis

Antimicrobial concentrations were compared between groups using the Kruskal–Wallis test, and paired comparisons of concentrations at baseline versus end of dwell time were analyzed with the Wilcoxon test. Statistical significance was set at an alpha level of 0.05 (two-tailed). Analyses were performed using SPSS software, version 15.0.1 (SPSS Inc., Chicago, IL, USA). Given the exploratory pharmacokinetic nature of the study, statistical analyses were primarily descriptive and hypothesis-generating.

ChatGPT (GPT-5.2, OpenAI) was used to assist in the development of the graphical abstract, following a preliminary concept and step-by-step guidance from the authors. The AI tool did not generate independent scientific content. The authors critically reviewed, modified, and validated the final graphical abstract.

## 5. Strengths and Limitations

### 5.1. Strengths

This study represents the first randomized, pharmacokinetic-driven clinical trial specifically designed to define optimal dwell times of antimicrobial lock solutions (ALSs) in totally implantable venous access ports, addressing a long-standing knowledge gap not covered by current guidelines.

Intraluminal antimicrobial concentrations were directly quantified in vivo under real clinical conditions, using standardized high-performance liquid chromatography with urea correction, providing robust and reproducible pharmacokinetic data that are rarely available in antimicrobial lock studies.

The simultaneous evaluation of multiple antimicrobials commonly used in clinical lock therapy (vancomycin, teicoplanin, linezolid, daptomycin, and tigecycline) allowed agent-specific characterization of concentration stability and replacement intervals, rather than extrapolating findings from a single compound.

The stepwise, dwell-time escalation design enabled identification of pharmacokinetic thresholds for loss of adequate intraluminal exposure, while minimizing patient risk and avoiding unnecessary prolongation of antimicrobial exposure, consistent with antimicrobial stewardship principles.

### 5.2. Limitations

Preservation of antimicrobial concentrations within the lock solution should not be interpreted as a surrogate for clinical efficacy. Although adequate and sustained intraluminal exposure is a necessary prerequisite for antimicrobial activity in lock therapy, particularly against planktonic organisms, mature biofilms display complex tolerance mechanisms that are not solely governed by pharmacokinetic factors. Accordingly, concentration stability alone cannot predict eradication success in established biofilm-associated infections.

The sample size within each antimicrobial–dwell time subgroup was limited (n = 5 per time point), reflecting the exploratory, pharmacokinetic nature of the study and limiting statistical power and generalizability.

The study was conducted exclusively in noninfected, recently implanted ports, in order to isolate intraluminal pharmacokinetic behavior under controlled conditions. This design choice may limit extrapolation to long-term devices or ports with established infection and mature biofilms, where additional biological and mechanical factors may influence antimicrobial performance.

The pharmacokinetic threshold used to define adequate intraluminal exposure (≥1 mg/mL) was based on pharmacokinetic/pharmacodynamic principles rather than validated clinical outcome data. Therefore, translation of these findings into real-world effectiveness should be approached with caution.

Differences in antimicrobial reconstitution solutions (normal saline versus lactated Ringer’s solution), dictated by formulation requirements, may have influenced chemical stability, as variations in pH and ionic composition could affect degradation or adsorption phenomena.

Only the initial 2 mL aspirate was analyzed for antimicrobial concentration, which may not fully capture potential intraluminal concentration gradients along the entire catheter length.

The planned 10-day dwell time arm could not be completed due to recruitment constraints, preventing definition of the upper limit of safe and effective dwell duration.

The findings are specific to the antimicrobial agents, concentrations, and catheter conditions evaluated in this study and may not be directly applicable to alternative formulations, catheter materials, or patient populations.

## 6. Clinical Implications

Antimicrobial lock solution (ALS) replacement intervals should be individualized according to the specific antimicrobial agent used, rather than applying uniform daily replacement strategies. A pharmacokinetic-informed approach allows optimization of intraluminal exposure while avoiding unnecessary catheter manipulation.

Vancomycin- and linezolid-based locks require daily replacement to maintain adequate intraluminal concentrations, whereas teicoplanin-, daptomycin-, and tigecycline-based locks preserve pharmacokinetically adequate concentrations for up to 7 days, supporting less frequent replacement in selected scenarios.

Extending ALS dwell times for pharmacokinetically stable agents has the potential to substantially reduce port manipulations, which may translate into lower procedure-related complications, reduced risk of mechanical contamination, and improved patient comfort, particularly in oncology and long-term catheter carriers.

From a healthcare system perspective, pharmacokinetic-guided ALS replacement strategies could reduce nursing workload, procedural burden, and overall costs, without compromising intraluminal antimicrobial exposure, thereby improving efficiency of care delivery.

Optimizing dwell time based on intraluminal pharmacokinetics may also contribute to antimicrobial stewardship efforts by limiting unnecessary antimicrobial handling and avoiding prolonged exposure to sub-inhibitory concentrations that could favor resistance selection.

These findings provide a practical framework to inform the design of future outcome-driven clinical trials, in which pharmacokinetically stable agents and rational replacement intervals can be prospectively evaluated for clinical efficacy, microbiological eradication, and catheter salvage.

## Figures and Tables

**Table 1:** Baseline characteristics of the study population by antimicrobial lock group.

Characteristic	Vancomycin (n = 10)	Teicoplanin (n = 20)	Linezolid (n = 10)	Daptomycin (n = 20)	Tigecycline (n = 20)
Sex, n (%)					
Male	7 (70)	12 (60)	6 (60)	14 (70)	8 (40)
Female	3 (30)	8 (40)	4 (40)	6 (30)	12 (60)
Age, years					
Mean	58.0	57.8	62.3	48.0	55.2
Median	61.8	62.9	64.2	52.7	55.1
Underlying malignancy, n (%)					
Solid tumor	10 (100)	19 (95)	7 (70)	15 (75)	17 (85)
Hematologic malignancy	0 (0)	1 (5)	3 (30)	5 (25)	3 (15)
Port implant site, n (%)					
Right subclavian vein	6 (60)	15 (75)	4 (40)	9 (45)	9 (45)
Right jugular vein	2 (20)	3 (15)	4 (40)	4 (20)	4 (20)
Other locations	2 (20)	2 (20)	2 (20)	7 (35)	7 (35)
Duration of catheterization prior to randomization, days					
Mean	21.6	42.1	11.4	39.5	42.2
Median	12.6	122.5	21.1	83.0	35.7

Note: Baseline demographic and clinical characteristics were broadly comparable across antimicrobial lock groups. Values are presented as number (percentage) or mean/median, as appropriate.

**Table 2:** Antimicrobial concentrations recovered from lock solutions after predefined dwell times.

Antimicrobial (Instilled Concentration)	1 day Mean (Median)	*p* Value ᵃ	3 days Mean (Median)	*p* Value ᵃ	5 days Mean (Median)	*p* Value ᵃ	7 days Mean (Median)	*p* Value ᵃ	Notes
Vancomycin (2 mg/mL)	1548.0 (1537.5)	0.80	646.7 (461.8)	0.04	—	—	—	—	Stopped after day 3
Teicoplanin (10 mg/mL)	6755.7 (7183.4)	0.04	6201.9 (5684.4)	0.04	7566.6 (7677.1)	0.13	10541.2 (9904.6)	0.89	
Linezolid (1.8 mg/mL)	886.1 (1032.5)	0.04	669.1 (727)	0.04	—	—	—	—	Stopped after day 3
Daptomycin (5 mg/mL)	4029.3 (4385.8)	0.04	2788.7 (2860)	0.04	2697.6 (2814.9)	0.04	2900.8 (2729.7)	0.04	
Tigecycline (4.5 mg/mL)	2405.5 (2415.9)	0.04	1441.8 (1402)	0.04	1092 (1180.4)	0.04	1101.1 (1062.6)	0.04	

Note: Values are expressed as mean (median) antimicrobial concentration recovered (mg/mL). ^a^ *p* values were calculated using the Wilcoxon signed-rank test comparing baseline and post-dwell concentrations.

## Data Availability

The data supporting the findings of this study are publicly available at ClinicalTrials.gov (https://clinicaltrials.gov) under the corresponding study identifier. No additional datasets were generated or analyzed beyond those reported in the registry.

## Clinical Trial Registration

EudraCT number 2010-023814-29. Trial Registration Number: Sponsor’s Protocol Code Number CAS110775. Registration Date: 04/11/2010.
